# Electroretinographic Evaluations of Eyes With Endophthalmitis

**DOI:** 10.1167/tvst.13.8.20

**Published:** 2024-08-12

**Authors:** Shunichiro Takano, Yuro Igawa, Yasuhito Narita, Takuhei Shoji, Yuji Yoshikawa, Takeshi Katsumoto, Tatsukata Kawagoe, Masayuki Shibuya, Jun Makita, Kei Shinoda

**Affiliations:** 1 Department of Ophthalmology, Saitama Medical University, Saitama, Japan; 2 Koedo Eye Institute, Saitama, Japan

**Keywords:** electroretinogram, endophthalmitis, photopic response, flicker response

## Abstract

**Purpose:**

To determine the physiological status of the retina of eyes with endophthalmitis by examining the electroretinograms (ERGs) recorded with a portable recording system and to determine whether the pretreatment ERG findings were correlated with the best-corrected visual acuity (BCVA) after the treatment.

**Methods:**

We examined the medical records of 118 eyes of 108 patients who were diagnosed and treated for infectious endophthalmitis at Saitama Medical University Hospital, Japan, between January 2015 to November 2022. Of these, we studied the 25 eyes of 21 patients who had been evaluated by electroretinography. In bilateral cases, one eye was analyzed. The eyes were classified into those with postoperative endophthalmitis (group S, n = 12) and those with endogenous endophthalmitis (group E, n = 9). Photopic and flicker ERGs were recorded with the RETeval system. The pretreatment clinical factors studied were the ERG components that might be correlated with the post-treatment BCVA.

**Results:**

Eyes in Group E with larger amplitude flicker ERGs (*P* = 0.0053, ρ = −0.8333) had better BCVA after treatment. In Group S, eyes with larger amplitude flicker ERGs (*P* = 0.0086, ρ = −0.7173), photopic a-waves (*P* = 0.0323, ρ = 0.6177), and photopic b-waves (*P* = 0.0055, ρ = −0.7443) had better BCVA after treatment.

**Conclusions:**

Simple and rapid ERG evaluations under light-adapted condition are helpful in evaluating the pretreatment retinal function and to determine the visual prognosis in eyes with endophthalmitis.

**Translational Relevance:**

Simple and non-time-consuming ERG evaluations are helpful in evaluating the retinal function in eyes with endophthalmitis and predicting the visual prognosis.

## Introduction

Endophthalmitis is the one of the most destructive infections of the eye that can result in poor visual outcomes within hours or days.^1^^–^^3^ Endophthalmitis is divided into exogenous and endogenous types. In the former, micro-organisms on the ocular surface or externally enter the eye to cause the endophthalmitis.^3^^–^^8^ The second type arises from a hematogenous dissemination during bacteremia or fungemia.^9^ The treatment options for endophthalmitis include systemic antibiotics, subconjunctival and intravitreal injections of antibiotics, or vitreous surgery, in addition to topical antibiotic eye drops.^1^^–^^3^ Because the inflammation caused by intraocular infection can spreads to the retina rapidly and cause severe visual dysfunction, immediate diagnosis and treatment including surgery are required. Categorization of the type of endophthalmitis helps to identify the underlying etiology and most likely the causative organism.^1^^–^^3^ However, it cannot detect the impairments of retinal function. When ophthalmoscopic evaluations are difficult because of turbid ocular media, the diagnosis is delayed, and decision for the treatment strategy is also delayed.

The physiological status of the retina can be determined by electroretinography (ERG) without fundus observations. Horio et al.^10^ reported that the combined findings of the b-/a-wave ratio of <1.0 and early onset of endophthalmitis within one week after an intraocular lens implantation suggested an infection by a highly virulent organism and poor prognosis. However, only limited information is available on the clinical use of the ERGs before the treatment of endophthalmitis.^10^^–^^12^ One of the reasons for this may be that ERG testing requires about an hour to complete, and an eye with an infection or an open wound may prevent the use of the contact lens electrode necessary for the ERG recordings.

The use of skin electrodes to record reliable ERGs has been well validated.^13^^–^^18^ The skin electrodes are noninvasive, which makes it possible to test eyes with Type S endophthalmitis.^19^^–^^21^ The skin electrodes do not contact the ocular surface, whereas other ERG electrodes tend to be noninvasive, but do contact the ocular surface. These properties of the skin electrodes prompted us to investigate whether ERG recordings using skin electrode would be of assistance in the management of endophthalmitis.

Thus the purpose of this study was to determine whether reliable ERGs can be recorded with skin electrodes from eyes with Type S and Type E endophthalmitis, and to determine whether the characteristics of the ERGs can be prognosticators of the BCVA after the successful treatment of the endophthalmitis.

## Methods

### Participants

This was a retrospective, cross-sectional study of patients who were diagnosed with endophthalmitis. The procedures used were approve by the Ethics Committee of the Saitama Medical University (Iruma, Japan, IRB, 2021-073), and they adhered to the tenets of the Declaration of Helsinki. Patients who were diagnosed with endophthalmitis after intraocular surgery or had endophthalmitis of endogenous origin and had ERG recordings at the Saitama Medical University Hospital from January 2015 to November 2022 were studied.

**Figure 1.**
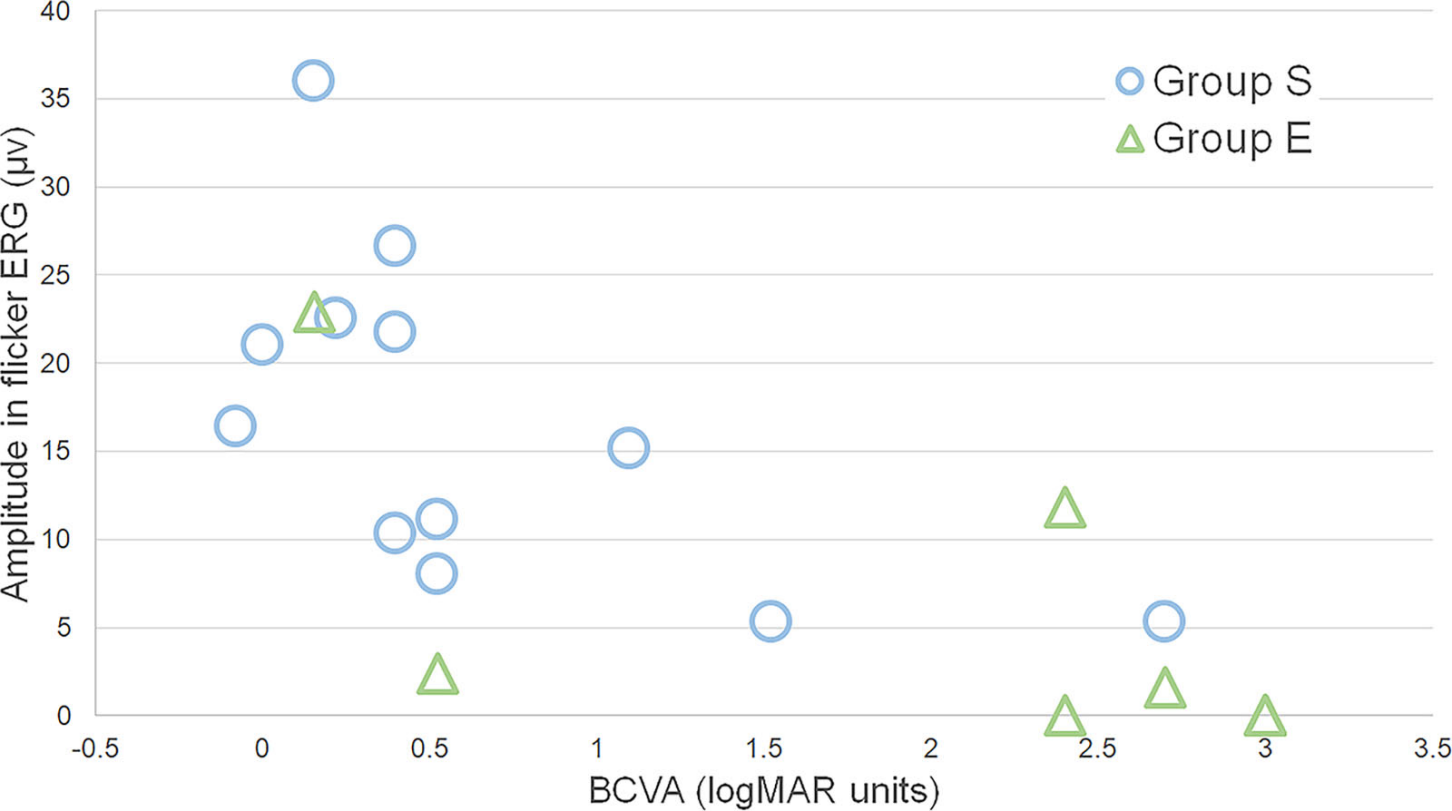
Relationship between the amplitude of the flicker ERGs and the visual prognosis in patients with exogenous and endogenous ophthalmitis. The amplitude of the flicker responses was significantly correlated with the BCVA after treatment in all eyes (*P* < 0.0001, ρ = −0.8614), in group S (*P* = 0.009, ρ = −0.717), and in group E (*P* = 0.005, ρ = −0.8333), Eyes with larger amplitude had better BCVA after treatment. Group E, endogenous endophthalmitis; Group S, postoperative endophthalmitis.

All patients had comprehensive ophthalmic examinations including measurements of the best-corrected visual acuity (BCVA), slit-lamp biomicroscopy, intraocular pressure measurements, and ophthalmoscopy. Patients with endophthalmitis associated with ocular trauma or corneal ulcer were excluded. There were four cases that developed endophthalmitis in both eyes, and in one case, one eye had no light perception at the initial examination; therefore only the other eye was included in the analyses. In the other three cases, only the eye that developed endophthalmitis first was studied. Only eyes where the BCVA before and after treatment and ERG recordings before treatment were available were studied. Ninety-five eyes of 84 patients had postoperative or endogenous endophthalmitis, and 21 eyes of 21 patients that met the inclusion criteria were studied (Fig. 1). The amplitudes and implicit times of the ERG components were measured by an embedded program, and their correlations with the decimal BCVA that was converted to the logarithm of the minimum angle of resolution (logMAR) was calculated.

### Full-Field ERG Recordings

Full-field ERGs were recorded using the RETeval system (LKC Technologies Inc, Gaithersburg, MD, USA), which is a portable ERG system that uses skin electrodes to pick up the ERGs. The pupils were dilated by topical 0.5% tropicamide and 0.5% phenylephrine hydrochloride, and the patient was adapted to the background light for 10 minutes before recording the photopic and flicker ERGs. Sensor strips of skin electrodes were carefully placed 2 mm below the lower eyelid margin after the skin was cleaned with an 80% ethanol solution. The electrodes were the active, reference, and ground electrodes. Although skin electrodes have traditionally not been used because of their lower signal levels,^22^ improvements in the hardware for data acquisition and Fourier-based analysis methods have achieved the reproducible results while avoiding the need to physically touch the surface of the eye with the electrodes.

A mini Ganzfeld dome was placed in front of the eye, and it had a red fixation LED to maintain the subject's fixation during the test. The device has an IR-sensitive camera and an IR LED to take videos of the eye with infrared light which enabled the examiner to monitor the eye position during the recording. The brief (<5 msec), 3 cd • s/m^2^ flashes on a 30 cd/m^2^ background was used to elicit the photopic ERGs. The stimulus frequency was 2 Hz, and 30 responses were automatically averaged. The flicker ERGs were elicited by the 3 cd • s/m^2^ flashes on a 30 cd/m^2^ background. The pulse duration was less than 1 msec, and the stimulus frequency was 28.3 Hz, and 147 to 421 responses were automatically averaged. The number summed depended on the signal-to-noise ratio of the ERGs. Signal acquisition was done with a sampling frequency of approximately 2 kHz. The light stimulation conditions conformed to the standard recommended by the International Society for Clinical Electrophysiology of Vision.^22^ The patients were instructed to look at the fixation point within the dome, and the patient's fixation was monitored by the infrared camera.

The implicit times and amplitudes of the a- and b-waves, and the flicker ERGs were automatically analyzed by the software embedded in the RETeval system. The a-wave amplitude was measured from the prestimulus baseline to the a-wave trough. The b-wave amplitude was measured from the a-wave trough to the b-wave peak. The implicit times of the a-wave and b-wave were measured from the onset of the stimulus to the peak of the wave. The amplitudes or magnitudes and implicit times of the fundamental component of the flicker ERGs were automatically measured and displayed by the RETeval system using a special algorithm of discrete Fourier transformation and cross-correlation analysis.^23^ Because the response to a periodic stimulus is composed of sinusoidal components that are multiples of the stimulus frequency, it is possible to reconstruct a less noisy version of the raw flicker ERG waveform by determining the amplitude and phase of each of the harmonics and summing them.^24^

### Statistical Analyses

We assessed the distribution of numerical variables by inspecting histograms and using the Shapiro-Wilk W-test of normality. The normally distributed variables are presented as the mean ± standard deviation (SD). Non-normally distributed variables and ordinal variables are presented as the medians and quartiles. The significance of the differences within the groups was determined by the Wilcoxon signed rank test and that between the groups was determined by the Wilcoxon rank sum test. The percentile distribution between groups was compared by Pearson's χ^2^ tests and Fisher's exact tests. The correlation between the values of the ERG components on the initial examination and post-treatment visual acuity was determined by Spearman's rank correlation coefficient. We analyzed the relationship between the BCVA after treatment and clinical factors such as age and BCVA before treatment, estimated duration before treatment, and ERG parameters before treatment. The decimal visual acuity was converted to the logarithm of the minimal angle of resolution (logMAR) units for statistical analysis. The visual acuities of “counting fingers,” “hand movements,” “light perception,” and “no light perception” were assigned values of 2.0, 2.4, 2.7, and 3.0 logMAR units, respectively.^25^ *P* values <0.05 were considered statistically significant. All statistical analyses were performed using JMP version 16 software (SAS Institute Inc., Cary, NC, USA).

## Results

### Demographics of Participants

The demographics of 21 eyes of 21 patients (15 men, six women, mean ± SD age 67.0 ± 14.7 years) are presented in Table 1. Twelve eyes of 12 patients with postoperative endophthalmitis were placed in Group S, and nine eyes of nine patients with endogenous endophthalmitis group were placed in Group E. Group S was composed of three eyes that developed endophthalmitis after cataract surgery, six eyes after vitrectomy, one eye after scleral buckling surgery with chandelier illumination, and two eyes after an intravitreal injection of antivascular endothelial growth factor agent. Group E was composed of three eyes of three patients with liver abscess, two eyes with pyelonephritis, one eye with prostatitis, one eye with periarticular abscess, and two eyes of unknown origin. Eight eyes were treated surgically. Pars plana vitrectomy was performed on 18 eyes (12 eyes in group S and six eyes in group E), and two eyes underwent enucleation (in E group). The two groups did not differ significantly in their characteristics except that the interval from onset of symptoms to treatment was significantly longer in Group E (*P* = 0.0009, Table 2).

**Table 1.** Clinical Characteristics of Patients With Endophthalmitis


Patients, Eyes	21
Group S	12
Group E	9
Sex	
Female	6 (28.6%)
Male	15 (71.4%)
Age (yrs), mean ± SD	67.0 ± 14.7
Surgical treatment was done, eyes	20
Pars plana vitrectomy was done, eyes	18
Group S	12
Group E	6
Underwent enucleation, eyes	2 (Group E only)
Combined cataract surgery with vitrectomy, eyes	6
Group S	1
Group E	5
Period from onset of symptoms to treatment (day), mean ± SD	
All eyes	6.0 ± 9.9
Group S	0.8 ± 1.0
Group E	12.9 ± 12.0
Group S vs. Group E (*P* value^*^)	0.0009
Causative pathogen, eyes^†^	5
Group S	2
Methicillin and quinolone-resistant *Staphylococcus epidermidis*	1
Staphylococcus (type unidentified)	1
Group E	3
*Streptococcus agalactiae*	1
Bacteria (type unidentified)	1
Fungus (type unidentified)	1

Group E, endogenous endophthalmitis; Group S, postoperative endophthalmitis.

* Mann-Whitney U test.

† The causative bacteria was detected by vitreous humor biopsy or aqueous humor biopsy.

In the other 16 eyes, the causative pathogen was unknown.

**Table 2.** LogMAR Visual Acuity in Eyes With Endophthalmitis

	Initial Visual Acuity	Post-Treatment Visual Acuity	*P* Value^*^
All eyes (n = 21)	2.1 (1.0, 2.4)	0.5 (0.4, 2.7)	0.0425
Group S (n = 12)	1.9 (0.8, 2.1)	0.4 (0.2, 0.5)	0.0059
Group E (n = 9)	2.4 (2.4, 2.7)	2.7 (2.4, 3.0)	1.0000
Group S vs. Group E (*P* value^†^)	0.0067	0.0073	

Values are shown as median (quartiles) of log MAR visual acuity.

Visual acuities of “counting fingers,” “hand movements,” and “light perception” were converted to 2.0, 2.4, and 2.7 logMAR units, respectively.

* Wilcoxon signed rank test.

† Mann-Whitney U test.

### Comparisons of BCVA and ERGs Between Groups S and E

The BCVA was significantly better in the eyes of Group S than Group E before (*P* = 0.0067) and after (*P* = 0.0073) the surgery (Table 2). The amplitude of the flicker ERGs (*P* = 0.0060), and the a- and b-waves of the photopic ERGs were significantly larger in Group S (*P* = 0.0024) than in Group E (*P* = 0.0019: Table 3). Representative responses from patients in Group S, Group E, and normal control are shown in the supplemental figure.^26^^,^^27^

**Table 3.** Electroretinographic Parameters Before Treatment in Eyes With Endophthalmitis

			Pretreatment LA 3.0 ERGs			
	Pretreatment Flicker ERGs		A-Wave		B-Wave	
	Amplitude (µV)	Implicit Time (ms)^*^	Amplitude (µV)	Implicit Time (ms)^*^	Amplitude (µV)	Implicit Time (ms)^*^
All eyes	10.3 (1.6, 21)	32.6 (28.8, 36.1)	−4.8 (−7.7, −0.38)	14.8 (13.7, 17.8)	14.1(1.1, 27.3)	34.2 (30.9, 36.1)
Group S	15.8 (9.7, 21.9)	32.0 (28.3, 34.8)	−7.1 (−8.8, −4.8)	14.9 (13.7, 15.9)	19.0 (15.6, 30.9)	33.2 (30.7, 35.4)
Group E	0 (0, 2.4)	38.1 (33.6, 42.5)	−0.26 (−0.68, 0)	14.3 (13.1, 17.8)	0 (0, 4.4)	36.5 (34.0, 40.6)
Group S vs. Group E (*P* value^†^)	0.0059	0.1016	0.0024	0.6348	0.0019	0.1631

Values are shown as median (quartiles).

* Eyes with distinguished electroretinogram were excluded from implicit analysis.

† Mann-Whitney U test.

### Correlation Between Pretreatment ERG Values and Prognosis in All Eyes

**Figure 2.**
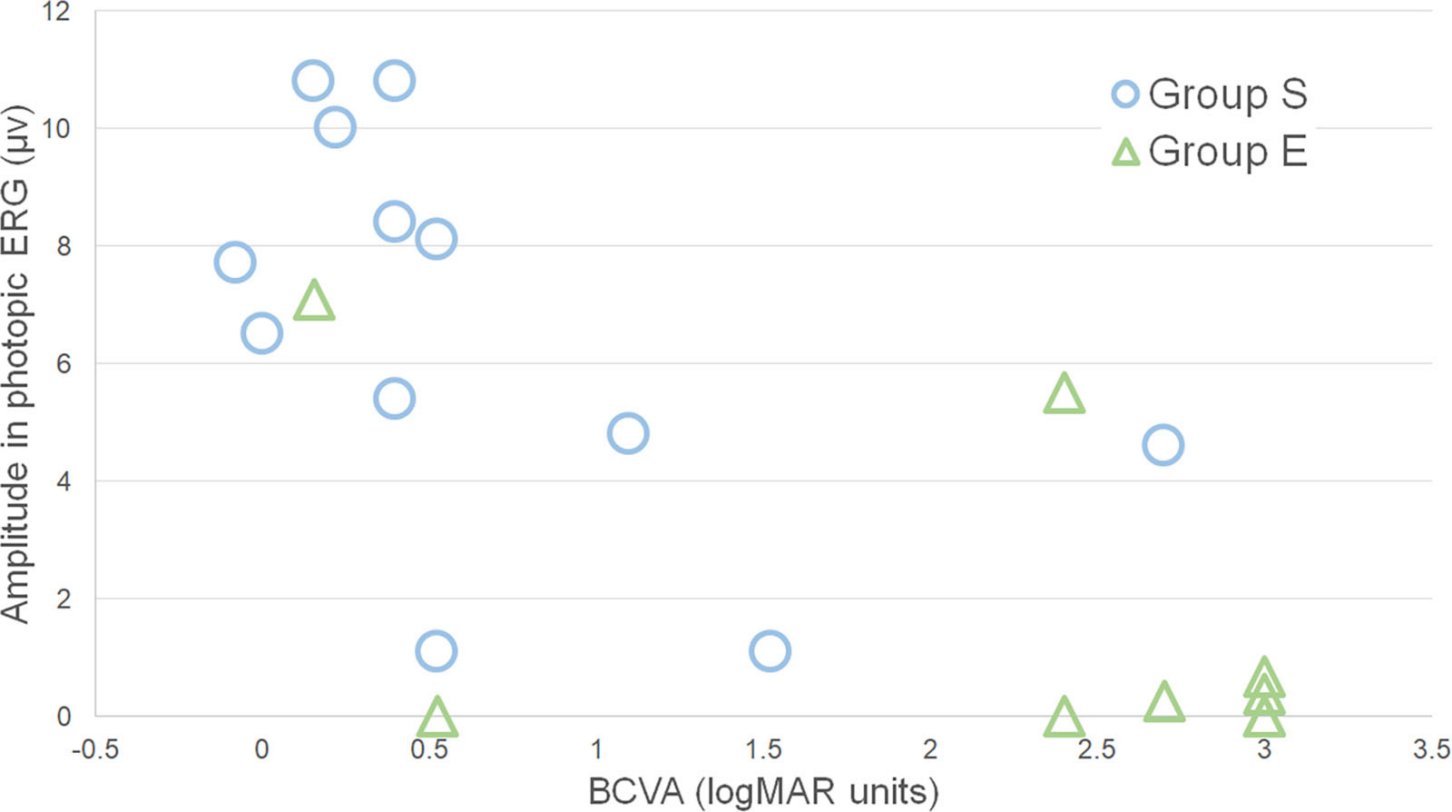
Relationship of the a-wave amplitude of the photopic ERGs with the visual prognosis. The amplitude of the photopic a-wave was significantly correlated with the BCVA after treatment in all eyes (*P* < 0.0001, ρ = 0.7590) and in group S (*P* = 0.0320, ρ = 0.6180). Eyes with larger amplitude had significantly better BCVA after treatment. On the other hand, the correlation was not significant in group E (*P* = 0.4988, ρ = 0.2603). Note that positive values are used for the a-wave amplitude so the relationships are in the same direction in all 3 figures.

**Figure 3.**
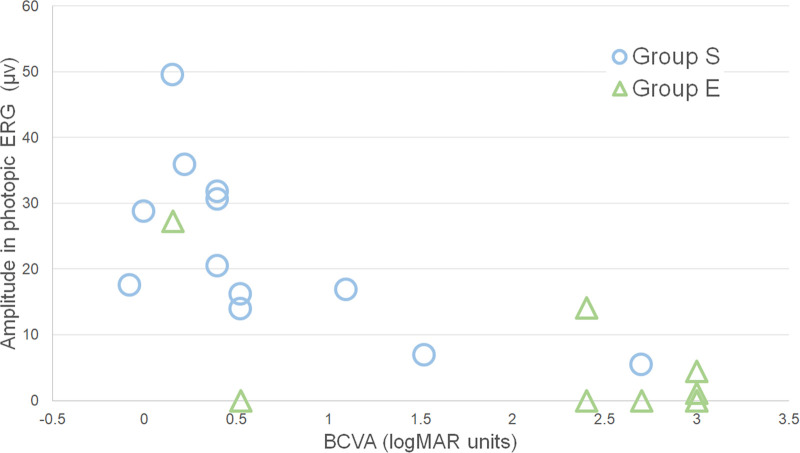
Relationship of the b-wave amplitude of photopic ERGs with visual prognosis. The amplitude of the photopic b-waves was positively and significantly correlated with BCVA after treatment in all eyes (*P* < 0.0001, ρ = −0.8180) and in group S (*P* = 0.0055, ρ = −0.7443). Eyes with larger amplitude had better visual acuity after treatment. On the other hand, the correlation was not significant in group E (*P* = 0.4853, ρ = 0.2682).

Our analyses showed that the interval from diagnosis to the time of treatment started (*P* = 0.0230, ρ = 0.4950) and the BCVA before treatment (*P* = 0.0004, ρ = 0.7043) were significantly correlated with the BCVA after the treatment. The amplitude of the flicker ERGs (*P* < 0.0001, ρ = −0.8614, Fig. 1), the photopic a-waves (*P* < 0.0001, ρ = 0.7594, Fig. 2), and the photopic b-waves (*P* < 0.0001, ρ = −0.8180, Fig. 3) were significantly correlated with the BCVA after the treatment. The implicit time of the flicker ERGs was correlated with the BCVA after the treatment (*P* = 0.0450, ρ = 0.5070). Eyes with larger amplitudes and shorter implicit times had better BCVA after treatment.

### Relationship Between Pretreatment ERG Values of Different Components and Prognosis in Groups S and E

In Group S, the BCVA before treatment was significantly correlated with the BCVA after the treatment (*P* = 0.0330, ρ = 0.6160), and eyes with larger amplitude flicker ERGs (*P* = 0.0090, ρ = −0.7173, Fig. 1), larger photopic a-waves (*P* = 0.0320, ρ = 0.6180, Fig. 2), and larger photopic b-waves (*P* = 0.0060, ρ = −0.7440, Fig. 3) had better BCVA after treatment. In Group E, only the amplitude of the flicker ERGs was significantly and negatively correlated with the post treatment BCVA (*P* = 0.0053, ρ = −0.8333; Fig. 1). This suggested that the larger the flicker ERG amplitudes was, the better was the visual prognosis.

## Discussion

The electrophysiological evaluations of the retina using a portable ERG recording system showed that several of the ERG parameters were significantly correlated with the post-treatment BCVA in eyes with both exogenous and endogenous endophthalmitis. Because intraocular infections can spread rapidly, prompt treatments are important to protect the retina. The RETeval photopic flicker ERG recording system has minimal risk of exacerbating the intraocular infection and requires a short period of time to perform. Thus it can assess the retinal function of eyes with endophthalmitis quickly and safely, and we recommend its use in patients with endophthalmitis.

In Group E, one-half of the eyes had non-recordable flicker ERGs, and the final BCVA was light perception or no light perception. On the other hand, eyes that had low amplitude flicker ERGs at the initial examination had a decimal BCVA ≤0.1. The reason why there were many flat ERGs in Group E is most likely because the endophthalmitis had affected the retina severely and the patient's general health was poor. The infection spreads from the blood circulation to the choroid and retina in endogenous endophthalmitis.^1^^,^^2^ When the fundus becomes difficult to see ophthalmoscopically, the ERGs were nonrecordable because the retinal outer layer was already affected.

There are only a few reports on the evaluations of retinal function by ERGs before treatment in eyes with endogenous endophthalmitis.^10^^,^^11^ Rowsey et al.^11^ classified the ERGs qualitatively into normal, abnormal, and flat, and they reported that patients with a markedly abnormal ERGs had poor recovery of the postoperative BCVA, whereas patients with near-normal ERGs had a better recovery of the BCVA. Our quantitative findings are in agreement.

Our results that the ERG findings were significantly correlated with the final BCVA even in cases with endogenous endophthalmitis suggest that the ERGs can be helpful in predicting the approximate prognosis. This is in line with previous reports.^10^^,^^11^

In Group S, the flicker ERG amplitudes, the photopic a- and b-wave amplitudes were significantly correlated with the BCVA after the treatment. Group S consisted of patients with postoperative endophthalmitis where the virulent organism invaded the anterior segment of the eye and spread to the retina. Thus in these cases the inflammation begins in the inner layers of the retina. This is supported by several studies of experimental endophthalmitis that showed that the b-wave amplitude was more reduced than the a-wave amplitude.^28^^–^^30^ Second, Horio et al.^10^ showed that the negative type of ERGs was a poor predictor of the visual outcomes in eyes with endophthalmitis. This agrees with the clinical experience that widespread vascular occlusion is often observed during vitreous surgery for acute exogenous endophthalmitis. Also, the flicker ERGs, which originates postreceptorally,^31^^,^^32^ may be susceptible to the inflammation that disseminates from the vitreous to the inner retinal layer. However, photopic ERGs were not best-suited for the comparisons of the inner and outer retinal layers. Although the ERGs with or without the negative type are optimal for comparing the degree of damage to the inner and outer retinal layers, it is observed only in the mixed rod and cone responses that requires a relatively long time for dark-adaptation. However, we believe that conventional ERGs do not fit with the clinical situation of endophthalmitis which requires rapid diagnosis and immediate treatment. Because our results showed significant correlations of not only the inner retinal responses but also the outer retinal responses with the visual prognosis, even photopic ERGs are useful to estimate the severity of the retinal impairment in eyes with endophthalmitis.

The RETeval system is a relatively new ERG recording system that uses skin electrodes and is thus less invasive.^16^ The RETeval flicker ERGs are simple, reproducible,^13^ and informative in selected situations to examine the retinal function in eyes with dense vitreous hemorrhage.^17^ It can also be used to diagnose the stage of diabetic retinopathy^33^ and determine the prognosis of central retinal vein occlusion.^30^ In addition, it has a low risk of exacerbating the condition of the eyes with open wounds or with infections. We have reported its feasibility for use after intraocular operations.^18^^–^^20^

There are limitations in this study. First, this study had a retrospective design and small sample size. These factors minimized the statistical power of trying to correlate the ERG parameters and the post treatment vision. Because the implicit times were not available in eyes with no ERG response, we performed statistical test only for the amplitudes. Furthermore, with a larger sample size, it may be possible to detect differences in the degree of correlation between the a- and b-waves with the visual prognosis. Further studies on a larger number of patients are needed to determine whether a layer-by-layer analysis of the ERGs can determine the specific retinal layer damaged by the endophthalmitis. A second limitation was that the underlying ocular disease and treatment were different. Because the underlying retinal disease and the therapeutic regimen may affect the ERG responses, it would be better to analyze the eyes with endophthalmitis after cataract surgery and those after vitrectomy separately and analysis on eyes that received the same treatment would be better. On the other hand, our study reflects the daily clinical situation and would be applicable to clinical practice. Third, we could not analyze ERG data recorded with conventional contact lens electrodes because it was not performed in any of our cohort. It would be of interest to compare the ERG responses recorded with skin electrode to those recorded with corneal contact electrode. Fourth, only the photopic ERGs were studied. The rod or mixed rod and cone ERGs would be more informative. However, the time needed for those ERGs may be critical in emergency cases. Comparisons between less time-consuming flicker ERGs and those more informative ERGs in an animal experiment will be interesting. In conclusion, simple and short time-consuming ERG evaluations under photopic conditions using hand-held recording system may be helpful in determining the visual prognosis as well as evaluating retinal function in eyes with endophthalmitis in an emergency.

## Supplementary Material

Supplement 1
