# Supplementary figures and images for: Electroretinographic Evaluations of Eyes With Endophthalmitis
Source: Transl Vis Sci Technol. 2024 Aug 12;13(8):20. doi: 10.1167/tvst.13.8.20 (PMC11323984; doi:10.1167/tvst.13.8.20)

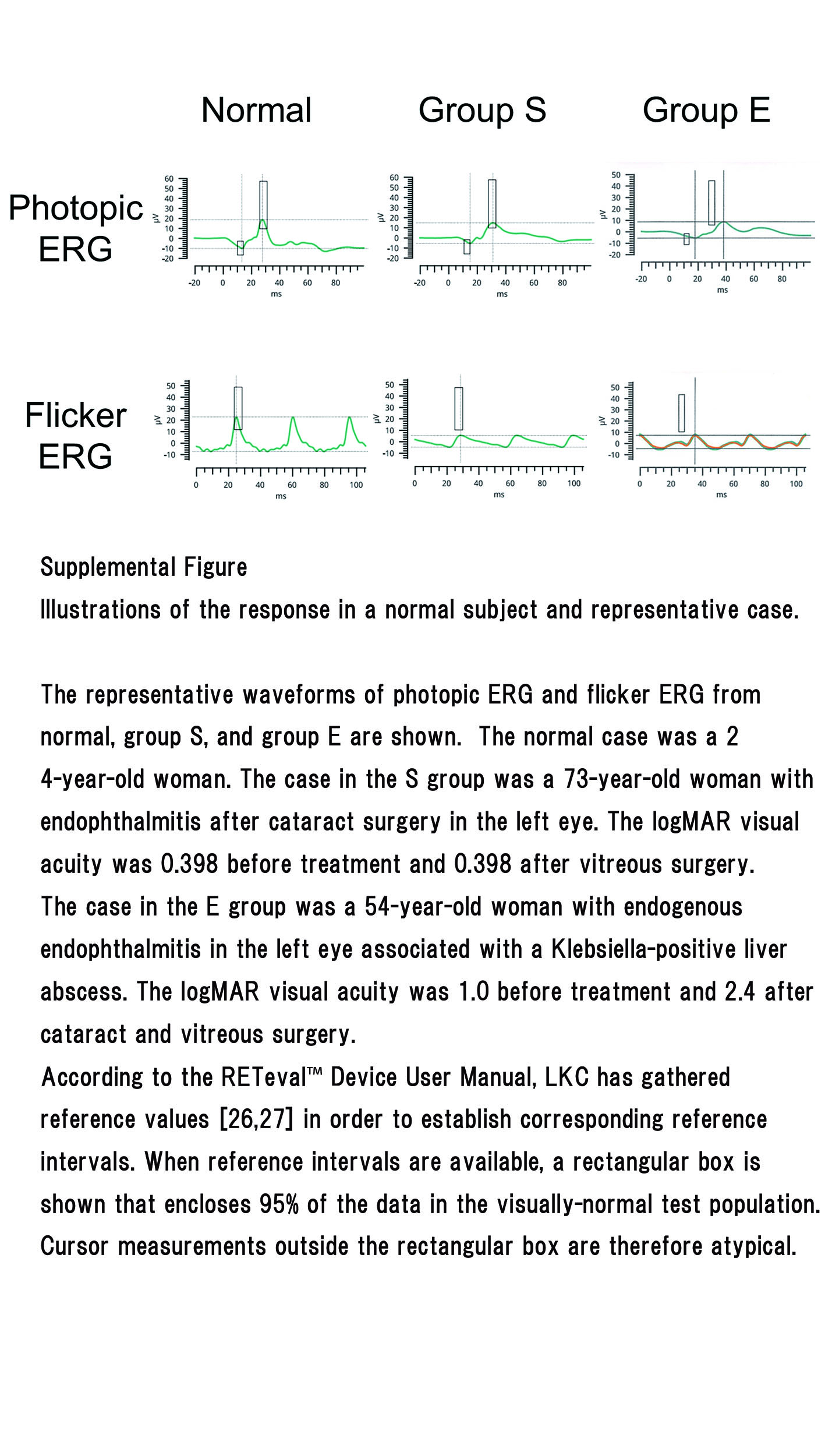

Supplement: Supplement 1 [file tvst-13-8-20_s001.jpg]
